# SYSTEMATIC REVIEW AND META-ANALYSIS OF RACIAL AND ETHNIC DIFFERENCES IN DEMENTIA CAREGIVER WELL-BEING

**DOI:** 10.1093/geroni/igz038.1624

**Published:** 2019-11-08

**Authors:** Chelsea Liu, Adrian Badana, Julia Burgdorf, Chanee D Fabius, William E Haley, David L Roth

**Affiliations:** 1 Johns Hopkins Bloomberg School of Public Health, Baltimore, Maryland, United States; 2 School of Aging Studies, University of South Florida, Tampa, Florida, United States; 3 Johns Hopkins University, Baltimore, Maryland, United States; 4 University of South Florida, Tampa, Florida, United States; 5 Johns Hopkins University School of Medicine, Baltimore, Maryland, United States

## Abstract

Studies comparing racial/ethnic differences on psychological and physical outcomes of dementia caregivers have often reported differences in well-being for minority groups compared to Whites. However, due to issues with enrolling minorities into studies, recruitment methods often differ for minority and White participants and may lead to biased comparisons. We conducted a systematic review and meta-analysis to examine racial/ethnic differences in dementia caregiver outcomes and to determine whether any differences vary among studies with population-based samples compared to convenience samples. We systematically reviewed articles with primary data from PubMed, Google Scholar and PsycINFO, and included studies comparing either African American (AA) or Hispanic/Latino dementia caregivers to White caregivers on measures of psychological health (e.g. depression, anxiety, burden) and physical health (e.g. self-rated health, cardiovascular measures, stress biomarkers). Reviewers screened titles and abstracts, reviewed full texts and conducted risk-of-bias assessments. A total of 207 effects were extracted from 40 studies. Random-effects models showed that Hispanics/Latinos reported significantly lower levels of well-being than Whites (ps < .05) for both psychological outcomes (37 effects) and physical outcomes (15 effects), while AAs were not significantly different from Whites in either domain. No differences were observed for population-based studies (N=3; 23 effects) or convenience-sample studies (N=37; 184 effects). Although some previous studies with convenience samples found better psychological well-being in AA caregivers, that pattern was not confirmed in our meta-analysis. Additional analyses for the different indicators of well-being and the relationship of quality ratings to effect sizes will be discussed along with implications for future research.

